# A novel cellular factor of *Nicotiana benthamiana* susceptibility to tobamovirus infection

**DOI:** 10.3389/fpls.2023.1224958

**Published:** 2023-07-18

**Authors:** Natalia Ershova, Kamila Kamarova, Ekaterina Sheshukova, Alexandra Antimonova, Tatiana Komarova

**Affiliations:** ^1^ Vavilov Institute of General Genetics, Russian Academy of Sciences, Moscow, Russia; ^2^ Belozersky Institute of Physico-Chemical Biology, Lomonosov Moscow State University, Moscow, Russia

**Keywords:** tobacco mosaic virus, Kunitz peptidase inhibitor-like protein (KPILP), virus susceptibility genes, intercellular movement, plant-virus interactions, proviral factor

## Abstract

Viral infection, which entails synthesis of viral proteins and active reproduction of the viral genome, effects significant changes in the functions of many intracellular systems in plants. Along with these processes, a virus has to suppress cellular defense to create favorable conditions for its successful systemic spread in a plant. The virus exploits various cellular factors of a permissive host modulating its metabolism as well as local and systemic transport of macromolecules and photoassimilates. The *Nicotiana benthamiana* stress-induced gene encoding Kunitz peptidase inhibitor-like protein (KPILP) has recently been shown to be involved in chloroplast retrograde signaling regulation and stimulation of intercellular transport of macromolecules. In this paper we demonstrate the key role of KPILP in the development of tobamovius infection. Systemic infection of *N. benthamiana* plants with tobacco mosaic virus (TMV) or the closely related crucifer-infecting tobamovirus (crTMV) induces a drastic increase in *KPILP* mRNA accumulation. *KPILP* knockdown significantly reduces the efficiency of TMV and crTMV intercellular transport and reproduction. Plants with *KPILP* silencing become partially resistant to tobamovirus infection. Therefore, KPILP could be regarded as a novel proviral factor in the development of TMV and crTMV infection in *N. benthamiana* plants.

## 1 Introduction

Due to a limited genome size and hence a limited protein encoding potential, the plant viruses resort to exploiting the cellular factors of their hosts at all stages of infection, creating favorable conditions both for intercellular and systemic transport throughout the plant. Moreover, plant viruses are capable of suppressing the antiviral defense mechanisms that are activated in response to viral infection (Wu et al., 1994; Wu and Shaw, 1996; Nelson and van Bel, 1998).

Tobacco mosaic virus (TMV) infection starts with a virion penetrating a plant cell, followed by an uncoating and synthesis of the non-structural proteins being essential for viral genome replication and transcription. To ensure a successful infection, three main processes are needed: viral RNA accumulation in the infected cells, intercellular spread of viral genetic material, and, finally, a long-distance transport (Heinlein, 2015; Ishibashi and Ishikawa, 2016). TMV intercellular spread is mediated by a non-structural 30 kDa movement protein (MP). MP interacts with viral and cellular factors, including plasmodesmata-associated proteins (PDAPs) (Ueki and Citovsky, 2014a; Dorokhov et al., 2019), in order to enable cell-to-cell movement of tobamoviral RNA (Liu and Nelson, 2013; Heinlein, 2015; Reagan and Burch-Smith, 2020; Sheshukova et al., 2020). MP binds viral RNA, facilitates its intracellular transport to plasmodesmata (PD) and affects PD permeability *inter alia*, via an indirect regulation of PD callose depositions (Amsbury et al., 2017). MP has been shown to interact with numerous host cell proteins, including the cytoskeletal proteins actin and myosin (Boyko et al., 2007; Guenoune-Gelbart et al., 2008; Hofmann et al., 2009; Amari et al., 2014) as well as myosin-binding protein (Kragler et al., 2003; Curin et al., 2007), cell wall pectin methylesterases (Dorokhov et al., 1999; Chen et al., 2000; Dorokhov et al., 2006), plasma membrane and PD proteins: synaptotagmins (Uchiyama et al., 2014; Yuan et al., 2018; Liu et al., 2020), remorins (Sasaki et al., 2018; Ma et al., 2022), ANK protein (Ueki and Citovsky, 2014b), calreticulin (Chen et al., 2005), etc.

In addition to MP, other TMV proteins were also found to be closely associated with the host factors. For example, TMV 126K component of the replicase was shown to interact with eukaryotic translation elongation factors 1A and 1B (Yamaji et al., 2010; Hwang et al., 2013). Moreover, replication complex components, 126K and 183K, were co-purified with chloroplast proteins, Rubisco activase (RCA) and ATP synthase γ-subunit (AtpC) (Bhat et al., 2013), and shown to interact with the *psbO*-encoded 33 kDa chloroplast protein, a component of the oxygen-evolving complex (Abbink et al., 2002). Another TMV component, the coat protein, was detected in association with chloroplast thylakoid membranes (Reinero and Beachy, 1986). Therefore, viral proteins interact with and exploit various cellular factors, inducing their structural and functional disturbance, thus suppressing or activating defense reactions (Bhattacharyya and Chakraborty, 2017; Rodriguez-Peña et al., 2021). Apart direct interactions between the virus and a host cell, there are multiple indirect effects of the viral infection. For instance, the chloroplast-resident DEAH-box RNA helicase, INCREASED SIZE EXCLUSION LIMIT2 (ISE2), has been implicated in virus-chloroplast interactions. Knockdown of *ISE2* in *Nicotiana benthamiana* plants leads to chlorosis development, activation of chloroplast retrograde signaling (CRS) and intercellular transport of macromolecules, as well as an increased sensitivity to TMV infection (Ganusova et al., 2017). At the same time, transient *ISE2* overexpression also resulted in susceptibility to TMV. The authors explain the increased sensitivity to TMV through the defense reactions activated via jasmonate signaling pathway that suppresses the salicylate-mediated resistance to the viral infection. Overall, these results in the susceptibility of tobacco plants to TMV (Ganusova et al., 2017). This example illustrates the subtle equilibrium between activation and suppression of plant antiviral defense mechanisms (Qiao et al., 2009; Bhat et al., 2013).

The above-mentioned cellular factors do not represent an exhaustive list. They are just a few examples of multiple interactions between the host cell factors and tobamoviral proteins that reflect a complex plant-virus interplay coupled with various ways the virus can exploit the plant cell. Most of these components are essential for the productive and effective viral infection, while not all of them are critical to cellular processes and cell viability. Consequently, these factors could be considered as genetic determinants of plant susceptibility to the virus (Garcia-Ruiz, 2018). The permissive host is characterized by the presence of all cellular factors ensuring a successful infection with a particular virus. Some of these genetic determinants are hardly detectable in the absence of viral infection, because their expression is induced only in response to a viral invasion or any other stress. Research into these virus-induced genes could broaden our knowledge about the plant-virus interaction and give us new opportunities for developing innovative techniques and approaches for antiviral crop protection.

We had previously identified *N. benthamiana* stress-induced gene encoding Kunitz peptidase inhibitor-like protein (KPILP) and showed that its expression is upregulated in response to GFP-encoding viral vector reproduction, prolonged darkness (Sheshukova et al., 2017a) and potato virus X (PVX) infection (Ershova et al., 2022). Recently, KPILP has been shown to be a positive regulator of intercellular macromolecular trafficking and implicated in the regulation of CRS (Ershova et al., 2022).

In this paper we explore the KPILP role in plant-virus interactions, specifically, KPILP functioning during a tobamoviral infection of *N. benthamiana* plants. We hypothesized that KPILP may be a plant susceptibility factor that is not essential for cellular “basic life support” under normal conditions, as it is activated only in response to different stress factors, which include viral infection as well. Viruses, inducing KPILP, could exploit it to modulate the metabolism and create favorable cellular environment for the viral infection. To test this hypothesis, we assessed the reproduction and local transport of GFP-encoding TMV- and crTMV-based viral vectors in plants with up- or downregulated *KPILP* expression and detected significant inhibition of viral intercellular spread, as well as decreased level of viral RNA accumulation in plants with suppressed *KPILP*. In addition, we analyzed the development of systemic TMV and crTMV infection in plants with decreased and increased *KPILP* expression. Symptom monitoring and viability registration during 40 days after inoculation with TMV or crTMV revealed that plants with *KPILP* knockdown acquired a partial resistance to tobamoviral infection, demonstrating less severe and delayed symptom development as well as an increased viability compared to plants with elevated *KPILP* expression. Taken together, these results indicate that KPILP may be a novel susceptibility factor of *N. benthamiana* to tobamovirus infection.

## 2 Materials and methods

### 2.1 Plant growth conditions

Wild type *Nicotiana benthamiana* plants were grown in the soil in a controlled environment chamber under a 16 h/8 h day/night cycle.

### 2.2 Agroinfiltration

*Agrobacterium tumefaciens* strain GV3101 was transformed with individual binary vectors and grown at 28°C in LB medium supplemented with 50 mg/l rifampicin, 25 mg/l gentamycin and 50 mg/l carbenicillin/kanamycin. *Agrobacterium* overnight culture was diluted with buffer containing 10 mM MES (pH 5.5) and 10 mM MgSO_4_, and adjusted to final OD_600_ of 0.01 for pPVX or pPVX(frKPILP) plasmids, OD_600_ of 0.3 for TMV:GFP and crTMV:GFP in the experiments with PVX-infected plants and OD_600_ 0.01 in the experiments with 35S-siKPILP. *Agrobacterium* suspension for pCambia1300 and 35S-siKPILP was diluted to OD_600_ of 0.1. Agroinfiltration was performed on almost fully expanded *N. benthamiana* leaves that were still attached to the intact plant. A bacterial suspension was infiltrated into the leaf tissue using a 2-ml syringe. After that the plants were incubated in greenhouse conditions.

### 2.3 Plant inoculation for systemic infection

*N. benthamiana* plants were inoculated with pPVX or pPVX(frKPILP) by agroinfiltration of the lower leaves, and in 10-14 days the systemic PVX infection was detected in the upper leaves. To induce TMV systemic infection, lower leaves of *N. benthamiana* plants were inoculated with 300 µg/ml suspension of virus particles in the presence of celite. To obtain plants with crTMV systemic infection, lower leaves were agroinfiltrated with the viral vector encoding crTMV infectious copy.

### 2.4 GFP imaging and quantification

GFP-containing foci of infection were visualized using a handheld UV lamp (λ = 366 nm). The foci area and fluorescence intensity were measured using open-source ImageJ software (Schneider et al., 2012).

### 2.5 Quantitative real-time PCR (qRT-PCR) analysis of transcript concentrations

Total RNA was extracted from plant tissues using the ExtractRNA reagent (Evrogen, Russia) according to the manufacturer’s instructions. For first strand cDNA synthesis, 0.1 mg of random hexamers and 0.1 mg of oligo-dT primer were added to 2 µg of total RNA, and reverse transcription was performed using Magnus reverse transcriptase (Evrogen, Russia) according to the manufacturer’s protocol. Quantitative real-time PCR was carried out using iCycler iQ real-time PCR detection system (Bio-Rad, Hercules, CA, USA). Reference genes were detected using the primers to 18S rRNA gene and protein phosphatase 2A gene (PP2A). The target genes were detected using sequence-specific primers and Eva Green master mix (Syntol, Russia) according to the manufacturer’s instructions. Primers used for qRT-PCR are listed in **Table S1**. Each sample was run three times, and non-template control was added to each run. A minimum of five biological replicates were performed. The results of qRT-PCR were evaluated using the Pfaffl algorithm (Pfaffl, 2001).

### 2.6 Plasmid constructs

To obtain 35S-siKPILP construct an approach based on the backbone of pKANNIBAL plasmid (Wesley et al., 2001) containing plant intron and multicloning sites for the insertion of sense and antisense fragment of the target sequence was used. 346-nt *KPILP* fragment (from 258 to 603 nt of the coding sequence) was amplified using the corresponding pair of primers to obtain two PCR products: the first (sense orientation) was flanked with XhoI and EcoRI recognition sites and the second (antisense orientation) – with BamHI and XbaI. Oligonucleotides used for PCR are listed in the **Table S1**. A fragment containing PDK intron was excised from pKANNIBAL using EcoRI and BamHI. At the next step, two abovementioned PCR products digested with the corresponding restriction enzymes (XhoI/EcoRI and BamHI/XbaI, respectively) together with PDK intron flanked with EcoRI/BamHI were ligated in pKANNIBAL plasmid digested with XhoI and XbaI. The obtained intermediate construct contained 35S promoter, sense KPILP fragment, PDK intron, antisense KPILP fragment and OCS terminator. This cassette was excised using PvuII restriction enzyme and transferred to pCambia1300 binary vector digested with PvuII resulting in final 35S-siKPILP construct.

### 2.7 Statistical analysis

The data was analyzed either by Student’s t-test or by one-way ANOVA as indicated in figure captions. The significance of difference between groups was assessed using Tukey honestly significant difference (HSD) test at *p* < 0.05 level or Student’s t-test. In all histograms, y-axis error bars represent the standard error of the mean values.

## 3 Results

### 3.1 *KPILP* mRNA accumulation drastically increases in response to TMV and crTMV infection

**Figure 1:**
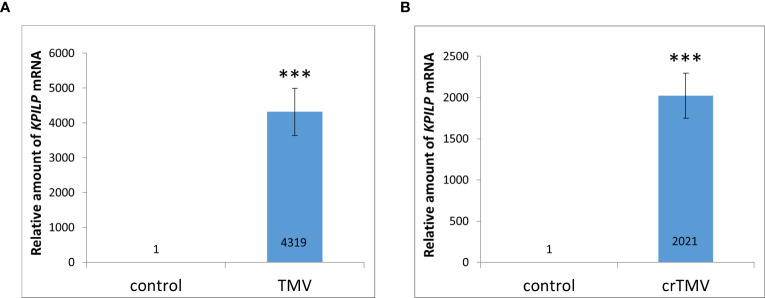
Systemic tobamovirus infection drastically stimulates *KPILP* expression. **(A, B)** The relative amount of *KPILP* mRNA in leaves with TMV **(A)** and crTMV **(B)** systemic infection as determined by qRT-PCR. The difference between the control (samples from the same plants before inoculation) and infected leaves is significant at p<0.001 (Student’s t-test) and marked with ***.

Previously, *KPILP* mRNA levels were shown to increase more than 100-fold during systemic TMV in *Nicotiana tabacum* and crTMV-based viral vector reproduction in *N. benthamiana* (Sheshukova et al., 2017a). Moreover, we have recently demonstrated that *KPILP* is induced in response to PVX infection. However, it increases only by a factor of 8-12 (Ershova et al., 2022). Here we used *N. benthamiana* as a model plant to study *KPILP* role during tobamovirus infection. First, we obtained plants with TMV and crTMV systemic infection confirmed by visible symptoms as well as viral RNA and coat protein accumulation detected in plant extracts (**Figure S1**) and analyzed *KPILP* mRNA levels in leaves with TMV (**Figure 1A**) and crTMV (**Figure 1B**) systemic infection. The results of qRT-PCR demonstrated that *KPILP* mRNA content was at least by 3 orders of magnitude higher in TMV- and crTMV-infected leaves as compared to the samples from the same plants before inoculation. This indicates that both TMV and crTMV systemic infection leads to a significant increase in *KPILP* expression.

However, such *KPILP* mRNA levels significantly exceed those obtained in experiments with crTMV-based viral vector lacking gene encoding coat protein (CP) (Sheshukova et al., 2017a). To check if this effect could be explained by the absence of one of the viral genes (*CP*) we performed agroinfiltration of *N. benthamiana* leaves with genetic constructs encoding either TMV CP or MP under control of 35S promoter and analyzed *KPILP* mRNA levels 3 days after agroinfiltration. We observed a ~10-fold increase in *KPILP* mRNA in response to *MP* expression and ~14-fold increase induced by *CP* (**Figure S2**). Thus, the individual viral genes do not stimulate *KPILP* expression in the same extent as systemic TMV and crTMV infection.

### 3.2 KPILP downregulates expression of nuclear genes encoding chloroplast proteins

**Figure 2:**
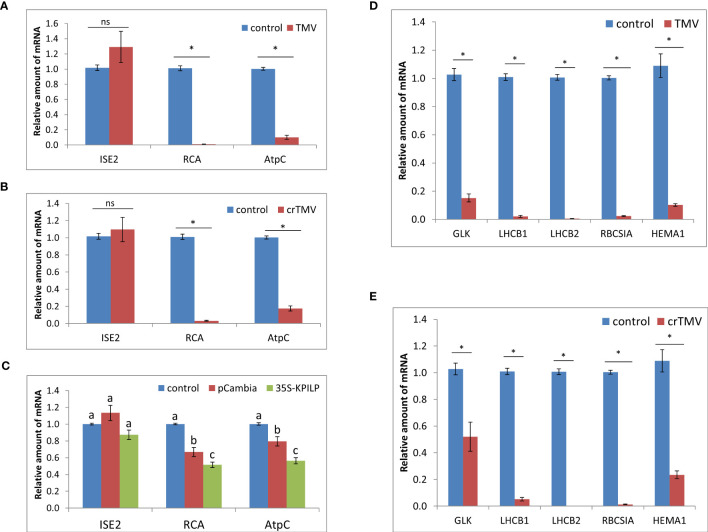
Tobamovirus infection downregulates photosynthesis-associated nuclear-encoded genes. Relative amount of mRNA in leaves with TMV **(A)** and crTMV **(B)** systemic infection, as revealed using qRT-PCR. Samples from the same plants before inoculation are taken as 1 (control), *, p<0.001; ns, not significantly different (Student’s t-test). **(C)** Relative amount of chloroplast protein encoding mRNA in leaves 3 days after agroinfiltration with “empty” pCambia1300 or 35S-KPILP as determined using qRT-PCR. Values for samples from non-infiltrated leaves are taken as 1 (control). Bars with different letters indicate significant difference at p<0.05 (ANOVA, Tukey HSD), while bars with the same letter are not significantly different. **(D, E)** Relative amount of CRS marker genes mRNA in response to TMV **(D)** or crTMV **(E)** infection as determined using qRT-PCR. The level of mRNA accumulation for each gene in leaves before inoculation was taken as 1. *, p<0.001 (Student’s t-test).

*N. tabacum* TMV infection results in a reduced expression of Rubisco activase gene, *RCA*, and ATP-synthase γ-subunit gene, *AtpC* (Sheshukova et al., 2017b). Both RCA and AtpC are host chloroplast factors that were co-purified with the viral replication complex during TMV infection. They are also important for ensuring a specific plant defense against tobamoviruses but not against PVX (Bhat et al., 2013). Analysis of *RCA* and *AtpC* mRNA levels during TMV and crTMV infection in *N. benthamiana* plants revealed their significant decrease (**Figures 2A, B**). Considering that *KPILP* was recently demonstrated to downregulate nuclear genes of chloroplast proteins which are regarded as CRS markers (Ershova et al., 2022), and *KPILP* mRNA accumulation showed more than a thousandfold increase in response to tobamovirus infection (**Figure 1**), we put forward that KPILP could affect *RCA* and *AtpC* genes expression. To test this hypothesis, we agroinfiltrated *N. benthamiana* leaves with 35S-KPILP and assessed *RCA* and *AtpC* mRNA levels in response to transient *KPILP* overexpression (**Figure 2C**). The results of qRT-PCR indicate that the increased *KPILP* expression leads to halving in *RCA* and *AtpC* mRNA accumulation. As an additional control, we used pCambia1300 binary vector, as agroinfiltration with and the “empty” vector *per se* slightly stimulates *KPILP* expression (Ershova et al., 2022). Indeed, we observed a 20-30% decline in *RCA* and *AtpC* mRNA levels in samples from pCambia1300 agroinfiltrated leaves (**Figure 2C**).

The nuclear-encoded chloroplast-localized RNA helicase *ISE2* is involved in chloroplast RNA processing and translation. Virus-induced silencing of *ISE2* results in a severe chloroplast dysfunction, suppresses chloroplast gene expression and activates CRS. At early stages of embryogenesis *ISE2* mutations are fatal to embryos (Burch-Smith and Zambryski, 2010; Burch-Smith et al., 2011). We checked whether the tobamovirus infection or *KPILP* overexpression induce any changes in *ISE2* mRNA levels. The results of qRT-PCR analysis revealed no significant difference between the control leaves and the leaves from TMV- (**Figure 2A**) and crTMV-infected (**Figure 2B**) plants or leaves with transient *KPILP* expression (**Figure 2C**). Therefore, the elevated *KPILP* expression induced either by tobamovirus infection or 35S-KPILP agroinfiltration did not affect *ISE2* mRNA accumulation.

To check whether the increase in KPILP, induced by tobamovirus infection affects the other chloroplast proteins encoded by nuclear genes including those associated with CRS, we assessed the expression of CRS marker genes encoding the components involved in the photosynthetic activity and defining the physiological status of chloroplasts: the transcriptional factor GOLDEN2-LIKE1 (GLK1) (Fitter et al., 2002); the light-harvesting complex antenna proteins LHCB 1 and 2; an isoform of rubisco small subunit (RBCS1A) (Bhat et al., 2013), the glutamyl-tRNA reductase protein (HEMA1) (Schmied et al., 2011). The qRT-PCR analysis revealed that expression of all these genes is downregulated during either TMV (**Figure 2D**) or crTMV (**Figure 2E**) systemic infection when *KPILP* expression is significantly increased (**Figure 1**).

### 3.3 KPILP stimulates reproduction and intercellular transport of TMV and crTMV

We have recently demonstrated that the increased *KPILP* expression facilitates cell-to-cell movement of 2xGFP reporter molecule and that KPILP *N*-glycosylation is indispensable for its ability to activate the intercellular transport. Moreover, it was shown that the upregulated *KPILP* is associated with decreased PD callose deposition (Ershova et al., 2022). To understand whether KPILP contributes to the viral intercellular movement, we used the previously developed model system for assessing KPILP-mediated effects. It is based on *KPILP* upregulation activated by PVX infection and *KPILP* suppression by virus-induced gene silencing (VIGS) using pPVX and pPVX(frKPILP) viral vectors, respectively (Ershova et al., 2022).

To estimate the efficiency of TMV or crTMV intercellular spread we used TMV:GFP and crTMV:GFP viral vectors, respectively, delivering the corresponding plasmids by agrobacteria into leaves of *N. benthamiana* plants with pPVX or pPVX(frKPILP) systemic infection and confirmed increased or suppressed *KPILP* expression. The control group contained intact plants of the same age. TMV:GFP and crTMV:GFP are capable of only local spread because of a lack of CP. Therefore, monitoring GFP-expressing foci of infection allows assessing the efficiency of infection and intercellular spread.

**Figure 3:**
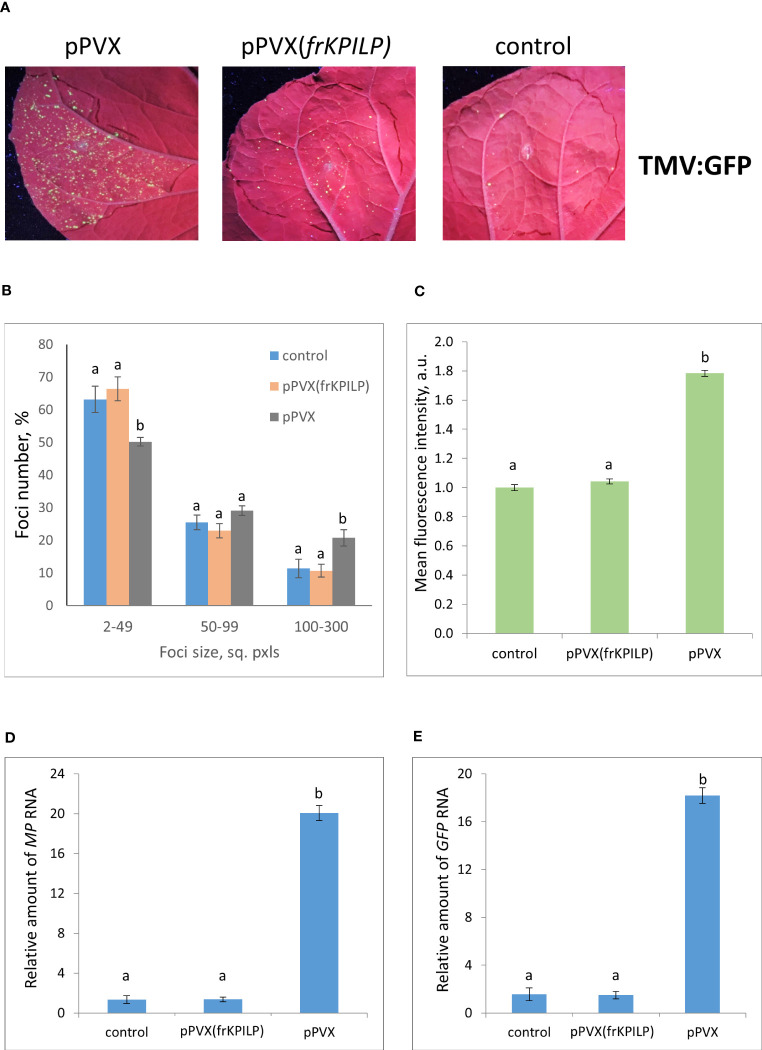
Upregulation of *KPILP* expression stimulates TMV:GFP reproduction and intercellular spread. **(A)** GFP-expressing foci visualization under UV light in *N. benthamiana* leaves of control plants (on the right) and plants with up- (on the left, pPVX) and downregulated [in the middle, pPVX(frKPILP)] *KPILP* expression 4 days after agroinfiltration with TMV:GFP. **(B)** Percentage of TMV:GFP-expressing foci of different size. **(C)** Mean GFP fluorescence intensity in analyzed foci. Relative amount of *MP* **(D)** and *GFP* **(E)** RNA in analyzed leaves quantified using qRT-PCR. Mean values and standard error are presented in histograms **(B–E)**. The data was analyzed using ANOVA. Bars without same letters indicate significant differences according to Tukey HSD at p<0.05.

**Figure 4:**
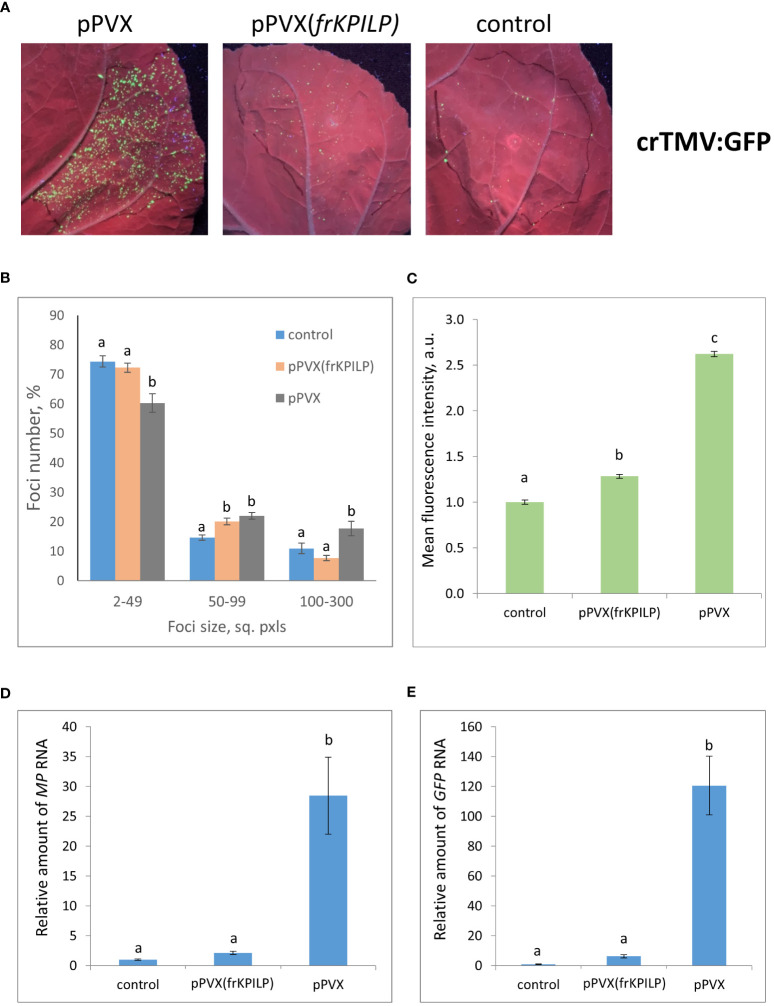
Upregulation of *KPILP* expression stimulates crTMV:GFP reproduction and intercellular spread. **(A)** GFP-expressing foci visualization under UV light in *N. benthamiana* leaves of control (on the right) plants and plants with up- (on the left, pPVX) and downregulated [in the middle, pPVX(frKPILP)] KPILP expression 4 days after agroinfiltration with crTMV:GFP. **(B)** Percentage of crTMV:GFP-expressing foci of different size. **(C)** Mean GFP fluorescence intensity in analyzed foci. Relative amount of *MP* **(D)** and *GFP* **(E)** RNA in analyzed leaves quantified by qRT-PCR. Mean values and standard error are presented in histograms **(B–E)**. The data was analyzed using ANOVA. Bars without similar letters indicate significant differences according to Tukey HSD at p<0.05.

To obtain the single cells transformed with either TMV:GFP or crTMV:GFP with further development of individual foci, we used the optimized dilutions of argobacterium suspensions. Results shown in **Figures 3**, **4** demonstrate that the GFP-expressing foci number is much lower in 4 days after agroinfiltration with either TMV:GFP (**Figure 3A**) or crTMV:GFP (**Figure 4A**) in control plants and plants with the suppressed *KPILP* expression compared to the plants with an increased *KPILP* level. Notably, *KPILP* mRNA accumulation level is comparable in the control plants and plants with *KPILP* VIGS induced by pPVX(frKPILP) infection (Ershova et al., 2022).

To assess the effect of KPILP on the efficiency of viral intercellular spread and reproduction we quantified the areas of GFP-expressing foci and the intensity of fluorescence in each experimental group. The percentage of larger foci (100-300 square pixels) is the highest in plants with upregulated *KPILP* expression, while the number of small foci (2-49 sq. pxls) is the lowest (**Figures 3B**, **4B**), indicating that the most effective intercellular transport of viral vectors is associated with elevated *KPILP* levels. GFP fluorescence intensity in each focus reflects the level of viral vector reproduction and GFP accumulation. The highest intensity is also observed in plants with upregulated *KPILP* (**Figures 3C**, **4C**).

Viral reproduction efficiency was additionally assessed using qRT-PCR of *MP* and *GFP* RNA in the infiltrated areas. The levels of the corresponding RNA in plants inoculated with TMV:GFP with the increased *KPILP* expression were about 20-fold higher than *MP* and *GFP* RNA levels in plants with silenced *KPILP* and control plants (**Figures 3D, E**). This indicates that viral reproduction is suppressed when *KPILP* is downregulated. Similar results were obtained in plants inoculated with crTMV:GFP (**Figures 4D, E**).

**Figure 5:**
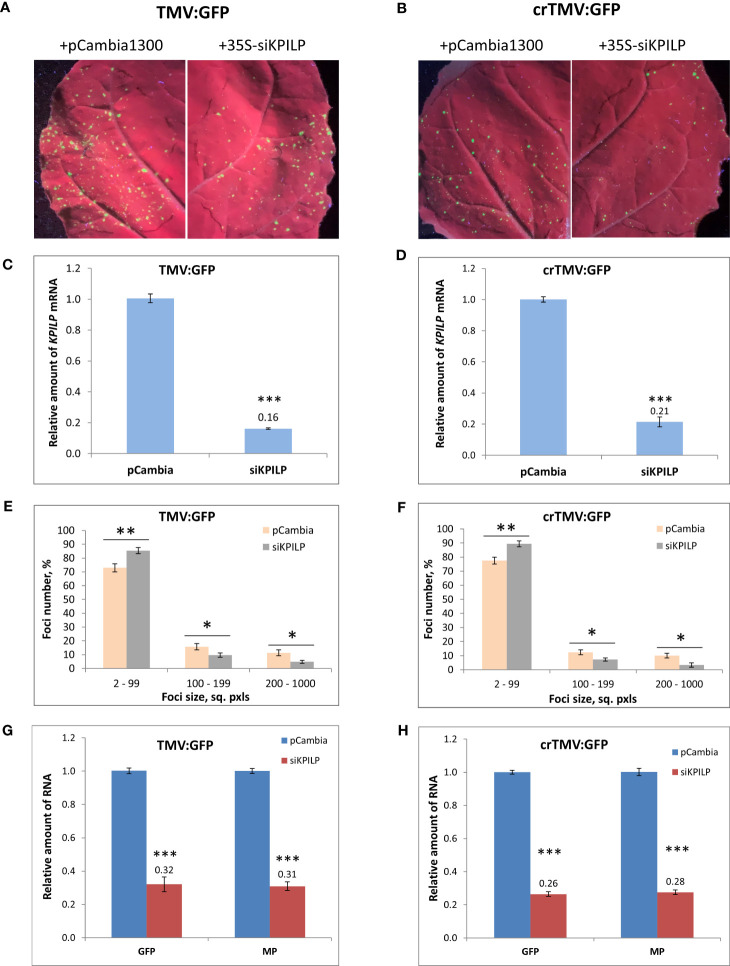
*KPILP* transient downregulation leads to the suppression of tobamovirus intercellular transport and reproduction. **(A, B)** GFP-expressing foci visualization under UV light in *N. benthamiana* leaves agroinfiltrated with TMV:GFP **(A)** or crTMV:GFP **(B)** together with 35S-siKPILP or pCambia1300. **(C, D)** *KPILP* expression 3 days after agroinfiltration in leaves shown in A and B, respectively. **(E, F)** Percentage of TMV:GFP- **(E)** and crTMV:GFP-expressing **(F)** foci of different size. **(G, H)** Relative amount of *MP* and *GFP* RNA in analyzed leaves quantified by qRT-PCR. Mean values and standard error are presented in histograms **(C–H)**. The data was analyzed using Student’s t-test. *, p<0.05; **, p<0.01; ***, p<0.001.

Plant agrobacterial transformation efficiency depends on numerous factors and represents a stress factor *per se*. In the abovementioned PVX-based system tobamovirus vectors were delivered via agroinfiltration when *KPILP* expression level was already premodified. Thus, it couldn’t be ruled out that this potentially led to the different efficiency of plant cells transformation by agrobacteria. To exclude the impact of this putative effect and additionally confirm that *KPILP* silencing negatively affects tobamovirus reproduction we used another experimental system. *KPILP* suppression was performed by the transiently expressed 35S-siKPILP cassette (**Figure S3A**) encoding 346-nt *KPILP* fragment in sense and antisense orientation separated by a plant intron. The intron was spliced in cells thus forming a hairpin RNA that induced *KPILP* silencing. TMV:GFP or crTMV:GFP vectors were introduced in *N. benthamiana* leaves simultaneously with 35S-siKPILP construct or pCambia1300 as a control (**Figures 5A, B**). 35S-siKPILP expression was confirmed by qRT-PCR (**Figures S3B, C**) and was shown to result in a 5-fold downregulation of endogenous *KPILP* levels (**Figures 5C, D**). An optimized agrobacterial suspension dilution allowed to obtain distinct areas of viral infection – *GFP*-expressing foci the size of which reflected the efficiency of TMV:GFP or crTMV:GFP intercellular spread. The results of foci size quantification (**Figures 5E, F**) indicate that *KPILP* silencing induced by the expression of 35S-siKPILP led to the increase by 10% of small foci (2-99 sq. pxls) number and the decrease of larger foci (200-1000 sq. pxls.) amount by 3-fold as compared to the control (viral vectors co-infiltrated with pCambia1300). We also assessed the level of viral RNAs produced from TMV:GFP or crTMV:GFP vectors and encoding MP and GFP in samples from the analyzed infiltrated areas. The results of qRT-PCR demonstrate 3-fold decrease in *MP* and *GFP* RNA accumulation for both viral vectors upon *KPILP* silencing compared to control (**Figures 5G, H**). Notably, the foci number for both TMV:GFP and crTMV:GFP halved when *KPILP* was downregulated compared to the control areas.

Therefore, we concluded that KPILP is essential for effective tobamovirus infection, reproduction, and intercellular transport.

### 3.4 *KPILP* silencing leads to increased *N. benthamiana* resistance to TMV and crTMV infection

**Figure 6:**
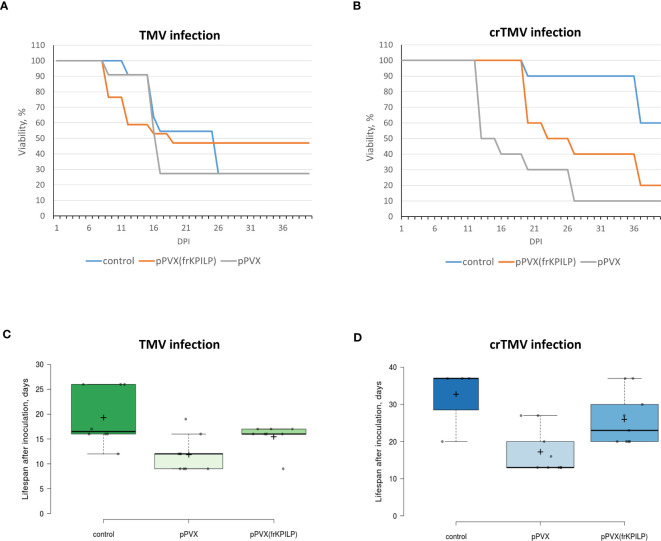
*KPILP* silencing leads to higher resistance of *N. benthamiana* to tobamovirus infection. **(A)** The viability of plants infected with TMV. **(B)** The viability of plants infected with crTMV. **(C)** The lifespan of plants with up- and downregulated *KPILP* inoculated with TMV. **(D)** The lifespan of plants with up- and downregulated *KPILP* inoculated with crTMV. In boxplots **(C, D)** central lines show the medians, and box boundaries indicate the 25th and 75th percentiles as determined by R software, whiskers extend 1.5 times the interquartile range from the 25th and 75th percentiles, outliers are represented by dots, crosses represent sample means, and data points are plotted as circles.

To assess KPILP role in the development of systemic tobamovirus infection, we used plants with up- and downregulated *KPILP* expression induced by pPVX or pPVX(frKPILP) vectors, respectively, and intact plants of the same age. All three groups were inoculated with TMV or crTMV to obtain the systemic infection. The experiment was repeated twice. We observed the development of symptoms characteristic for tobamovirus infection (**Figure S1A**) such as lesions on leaves, stem and petiole decay as well as wilting in all groups of plants. Importantly, the systemic TMV and crTMV infection commonly mortal *N. benthamiana* plants, unlike *N. tabacum* plants. During 40 days of monitoring the inoculated plants we documented all changes in the plant appearance and the time of their death. The death ratio of the infected plants by the 40^th^ day is presented in **Figure 6**. *KPILP* silencing decreases the death rate of TMV-infected plants compared to either the control group or to plants with elevated *KPILP* levels (**Figure 6A**). However, crTMV infection is not so sensitive to the lack of *KPILP*: 80% of the plants from the *KPILP*-silenced experimental group died by the 40th day after inoculation (**Figure 6B**). The survivors from the group with downregulated *KPILP* looked the same as plants from the control group (**Figure S4**). Another parameter that we analyzed was the mean lifespan of plants in each group. Most of the plants with upregulated *KPILP* died in two weeks after TMV inoculation, while plants with silenced *KPILP* had slightly longer lifespan (**Figure 6C**). In case of crTMV infection, we observed a significant and marked increase in the lifespan of plants with *KPILP* downregulation compared to plants with increased the *KPILP* level (**Figure 6D**). The results indicate that *KPILP* upregulation induced by pPVX vector shortens the plants’ lifespan after inoculation with TMV (**Figure 6C**) or crTMV (**Figure 6D**) compared to the control group, where *KPILP* was not upregulated before tobamovirus infection. Plants with silenced *KPILP* demonstrated higher resistance to TMV and crTMV infection compared to plants with increased *KPILP* expression. We concluded that elevated *KPILP* expression stimulates the development of tobamovirus infection and viral reproduction increasing the severity of symptoms, while *KPILP* suppression results in partial resistance to both TMV and crTMV infection.

## 4 Discussion

Plant viruses exploit a variety of strategies to successfully infect and spread throughout a plant. In this work we have explored the interaction between tobamoviruses and *N. benthamiana*, where the *KPILP* gene plays a regulatory and a potentially decisive role. In mature intact leaves, *KPILP* expression is suppressed. However, when the leaf tissues are infected with TMV, there is a sharp increase in its expression. In the previous paper (Sheshukova et al., 2017a) it was shown that the level of *KPILP* mRNA in roots is much higher than in mature and photosynthetically active leaves. The same paper demonstrated that in a TMV-infected tobacco leaf with mosaic symptoms, the light green zones with altered pigmentation and active virus replication exhibit a considerably higher level of *KPILP* mRNA than in dark green zones with normal pigmentation. This may implicate an inverse correlation between photosynthetic activity of chloroplasts and *KPILP* mRNA levels: (1) *KPILP* expression is active in roots where no photosynthesis occurs; (2) TMV affects functioning of chloroplast probably via activating the *KPILP* expression.

In this paper we demonstrated that the nuclear-encoded chloroplast RNA helicase *ISE2* mRNA accumulation in leaves with systemic tobamovirus infection remained at the same level as before infection. However, it was previously shown that *ISE2* expression increases at early stages of TMV infection by 18 hours after inoculation, and then its level decreases (Ganusova et al., 2017). We could not rule out that *ISE2* expression also changes in our system at earlier stages of infection but then it returns to the initial levels. In the same paper (Ganusova et al., 2017) it was demonstrated that sensitivity to TMV infection increases both in case of transient *ISE2* downregulation and constitutive overexpression. This means that *ISE2* plays an important regulatory role in plant defense response mediated by chloroplast signals. However, we did not find any significant correlation between *ISE2* mRNA levels and increased *KPILP* expression either activated by viral infection or during transient overexpression.

Nevertheless, it was shown that the elevated *KPILP* expression during tobamovirus infection is associated with suppression of genes important for chloroplast functioning including CRS marker genes *LHCB1*, *2*, *RBCSIA* and *HEMA1*. It is in line with the previous results demonstrating KPILP regulatory function toward the above-mentioned CRS marker genes in the transient expression system and during PVX infection (Ershova et al., 2022).

The expression of two other genes encoding chloroplast proteins – RCA and AtpC – was earlier demonstrated to be suppressed during TMV infection in *N. tabacum* but not in response to PVX. The virus-induced silencing of *RCA* and *AtpC* leads to a more efficient TMV spread and accumulation in *N. benthamiana*, which in turn determines the role of these factors in mediating antiviral responses, especially against tobamoviruses (Bhat et al., 2013). In this work we assessed the level of *RCA* and *AtpC* mRNA accumulation during the systemic tobamovirus infection and transient *KPILP* overexpression in *N. benthamiana* leaves. The systemic tobamovirus infection leads to a considerable suppression of these genes, which is in line with the previously published papers (Bhat et al., 2013; Ganusova et al., 2017; Sheshukova et al., 2017b). We also showed that transient *KPILP* expression halved the levels of *RCA* and *AtpC* mRNA in the intact leaves. The obtained results indicate that KPILP plays an important role in *RCA* and *AtpC* genes expression regulation as well as in regulation of the other examined photosynthesis-associated nuclear-encoded genes during tobamovirus infection.

Suggesting that KPILP could be a proviral host factor exploited by the virus for infecting a plant, we studied how *KPILP* expression affects local spread and reproduction of TMV and crTMV, using TMV:GFP and crTMV:GFP viral vectors, respectively. Inoculating leaves with these vectors enables the quantitative assessment of intercellular transport of the model viral vector by measuring the size of GFP-expressing foci of infection. The intensity of GFP fluorescence in the analyzed foci and the level of *MP* and *GFP* mRNA accumulation reflect the efficiency of virus reproduction.

The obtained results (**Figures 3**, **4**) indicated that there is a positive correlation between *KPILP* expression level and tobamovirus infection efficiency: in plants with upregulated *KPILP* we observed more active local spread of both tobamoviral vectors and higher levels of reproduction while in control group and plants with downregulated *KPILP* expression the viral intercellular transport and reproduction were significantly less efficient. Noteworthy, in this model system the *KPILP* level was modulated prior to agroinfiltration with TMV:GFP or crTMV:GFP. Thus, to properly interpret the obtained results and exclude the influence of *KPILP* on plant cell agrobacterial transformation another experimental set-up was utilized: the simultaneous delivery of plasmid encoding viral vector and the 35S-siKPILP construct that induces *KPILP* silencing. KPILP suppression in these experiments also led to a decreased efficiency of viral vectors’ reproduction and intercellular spread (**Figure 5**) as well as reducing the number of foci of infection. Moreover, using a different experimental set-up we confirmed that KPILP doesn’t affect the delivery of genetic material by agrobacteria but has a specific effect on the virus. Together these results allow us to conclude that KPILP plays an important role in tobamovirus infection development.

Using the system with up- and downregulated *KPILP* expression induced by pPVX or pPVX(frKPILP) vectors (Ershova et al., 2022), we assessed the sensitivity of the model plants to the development of systemic tobamovirus infection. We monitored the time in which the most severe symptoms developed, lifespan and viability of plants. **Figure 6** shows that plants where *KPILP* is activated have an increased sensitivity to the infection. This manifests itself in a shorter lifespan and a reduced ratio of the survivors compared to the group of plants with *KPILP* knockdown or the control group. Plants with the downregulated *KPILP* demonstrated an increased resistance to TMV and crTMV infection: the symptoms were less severe, lifespan was longer and the mortality percentage by the 40^th^ day after inoculation was lower. Monitoring the development of infection, we have not observed any significant difference between TMV- and crTMV-infected plants: the symptoms were similar. Moreover, plants with suppressed *KPILP* had similar appearance as the plants from the control group (**Figure S4**).

In a permissive host, a rapid tobamovirus infection development is possible only in case of favorable conditions for all the infection stages, from virion uncoating to penetration to the vascular system. TMV reaches the vasculature in 16-18 hours after infection (Nilsson-Tillgren et al., 1969). Such a rapid spread within the plant can occur only if the following events are properly synchronized: efficient viral genome expression and replication, synthesis of viral proteins, antiviral response suppression and intercellular transport activation. We can assume that the initial activation of cellular factors exploited by the virus could be induced by the coat protein (CP) which is the first to enter a cell in case of infection as a component of virion, or MP the small amounts of which could be synthesized directly on the template of TMV or crTMV genomic RNA. This remarkable feature of MP was shown for TMV and crTMV (Dorokhov et al., 1993; Dorokhov et al., 1994; Skulachev et al., 1999), whose genome contains internal ribosome entry site (IRES) (IRES_MP,75_^U1^, and IRES_MP,75_^CR^, respectively) mediating MP translation directly form genomic RNA.

We could speculate that MP and/or CP can potentially induce *KPILP* expression at the initial stages of infection directly or via other cellular factors. However, *MP* or *CP* overexpression *per se* does not lead to the 1000-fold increase in *KPILP* mRNA accumulation (**Figure S2**) as it happens in response to systemic TMV infection (**Figure 1**). Therefore, we could suggest that only in presence of all viral components *KPILP* expression is drastically activated due to the generalized effect of viral reproduction on all cellular components and their functioning. We hypothesize that upon TMV infection we observe a synergetic effect. And at the early steps of infection development KPILP launches irreversible changes in the photosynthetic apparatus and activation of intercellular transport. Although KPILP was not detected in chloroplasts, there is a clear inverse correlation between photosynthesis and *KPILP* expression activated during TMV infection. Moreover, TMV might exploit KPILP for suppression chloroplast activity, thereby influencing the antiviral response and regulating the cell-to-cell and long-distance transport.

Based on the obtained results we could regard KPILP as a proviral cellular factor and one of the susceptibility genetic determinants which is “dormant” in an aerial parts of the intact plant but activated by the viral infection. The question remains unanswered as to whether TMV has a direct effect on *KPILP* expression, suppressing in this way the functional activity of chloroplasts, or whether there are other proteins involved in this regulatory pathway. Therefore, search of potential KPILP partners or cellular factors upstream and downstream of KPILP-based cascade could elucidate the other participants in this interplay and is a subject of further investigation.

## Data availability statement

The original contributions presented in the study are included in the article/**Supplementary Material**. Further inquiries can be directed to the corresponding author.

## Author contributions

Conceptualization, NE and TK; design of the experiments and investigation, NE, KK, ES, and AA; data analysis, NE, KK, ES, and TK; writing—original draft preparation, NE and TK; writing—review and editing, NE and TK; funding acquisition, TK. All authors contributed to the article and approved the submitted version.
