# Supplementary figures and images for: A novel cellular factor of Nicotiana benthamiana susceptibility to tobamovirus infection
Source: Front Plant Sci. 2023 Jul 18;14:1224958. doi: 10.3389/fpls.2023.1224958 (PMC10390835; doi:10.3389/fpls.2023.1224958)

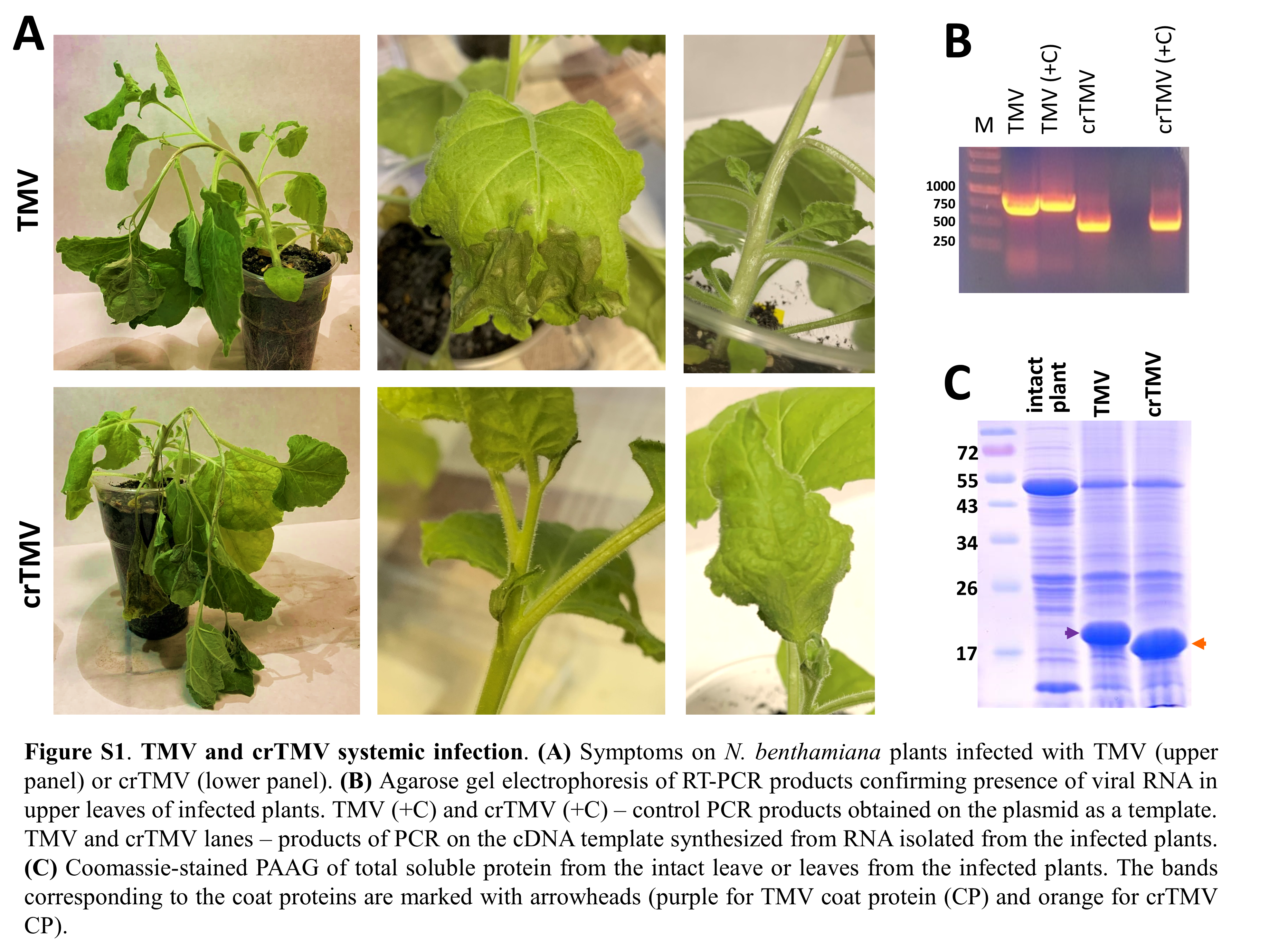

Supplement: Supplementary file 1 [file Image_1.tif]

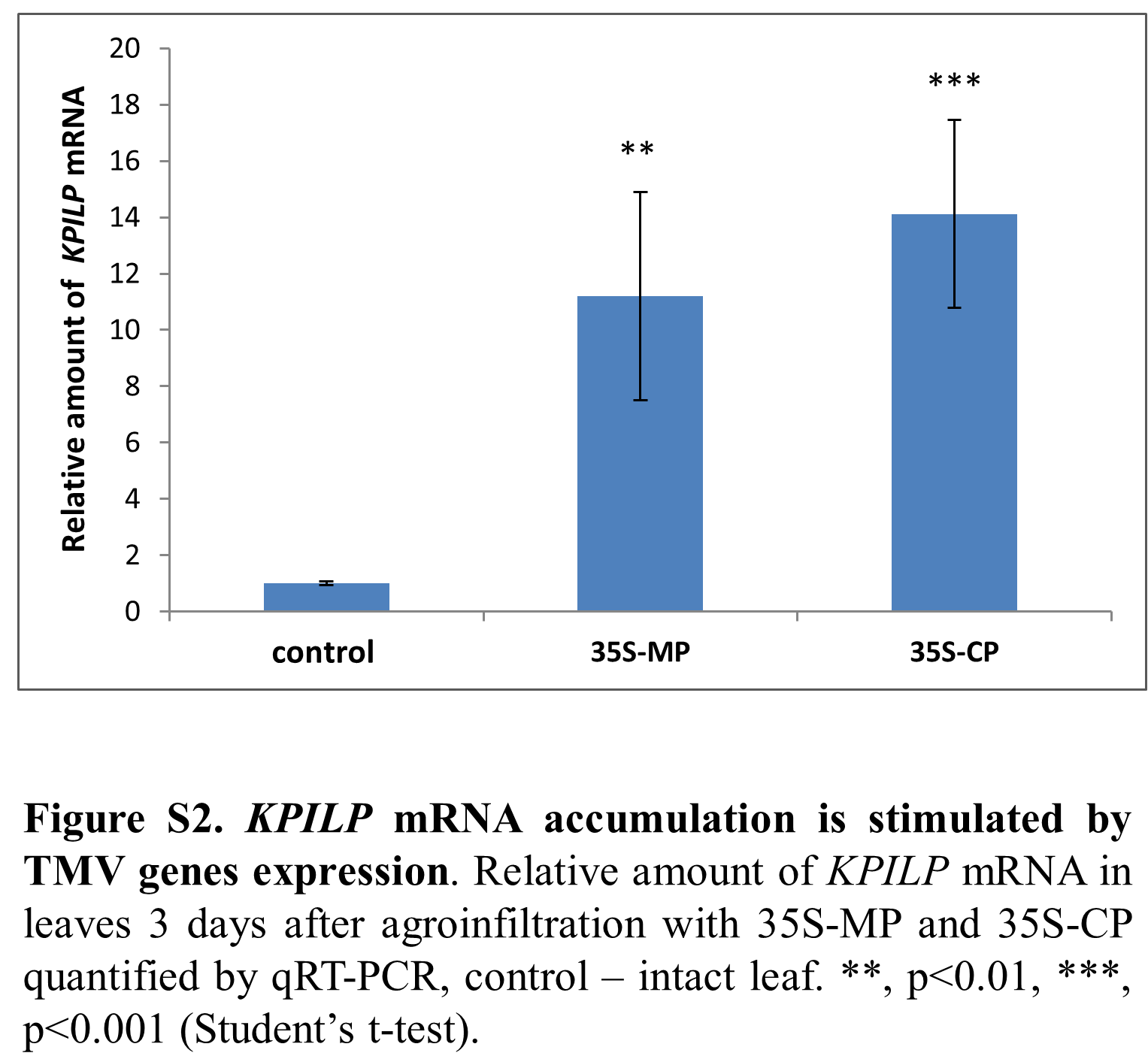

Supplement: Supplementary file 2 [file Image_2.tif]

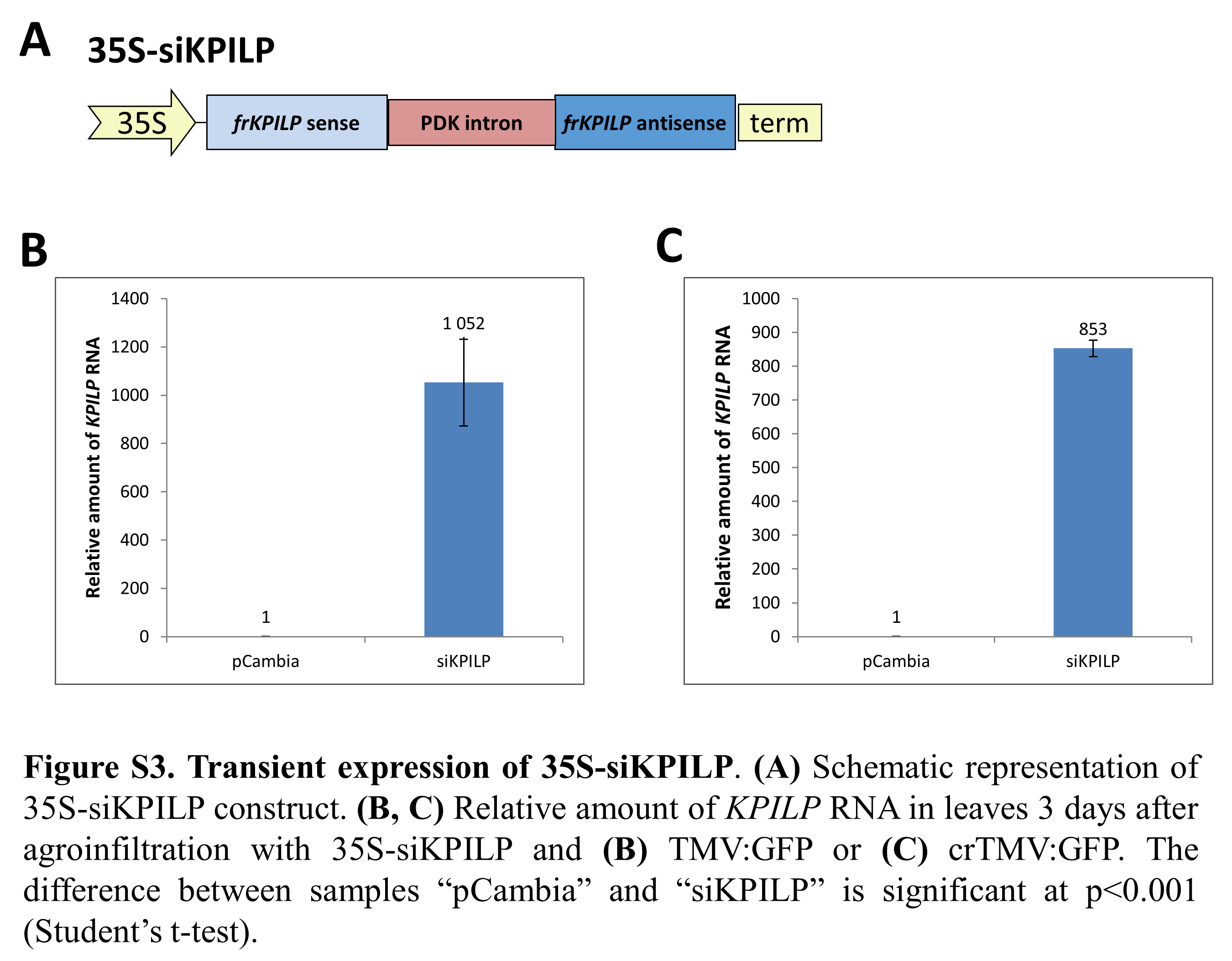

Supplement: Supplementary file 3 [file Image_3.tif]

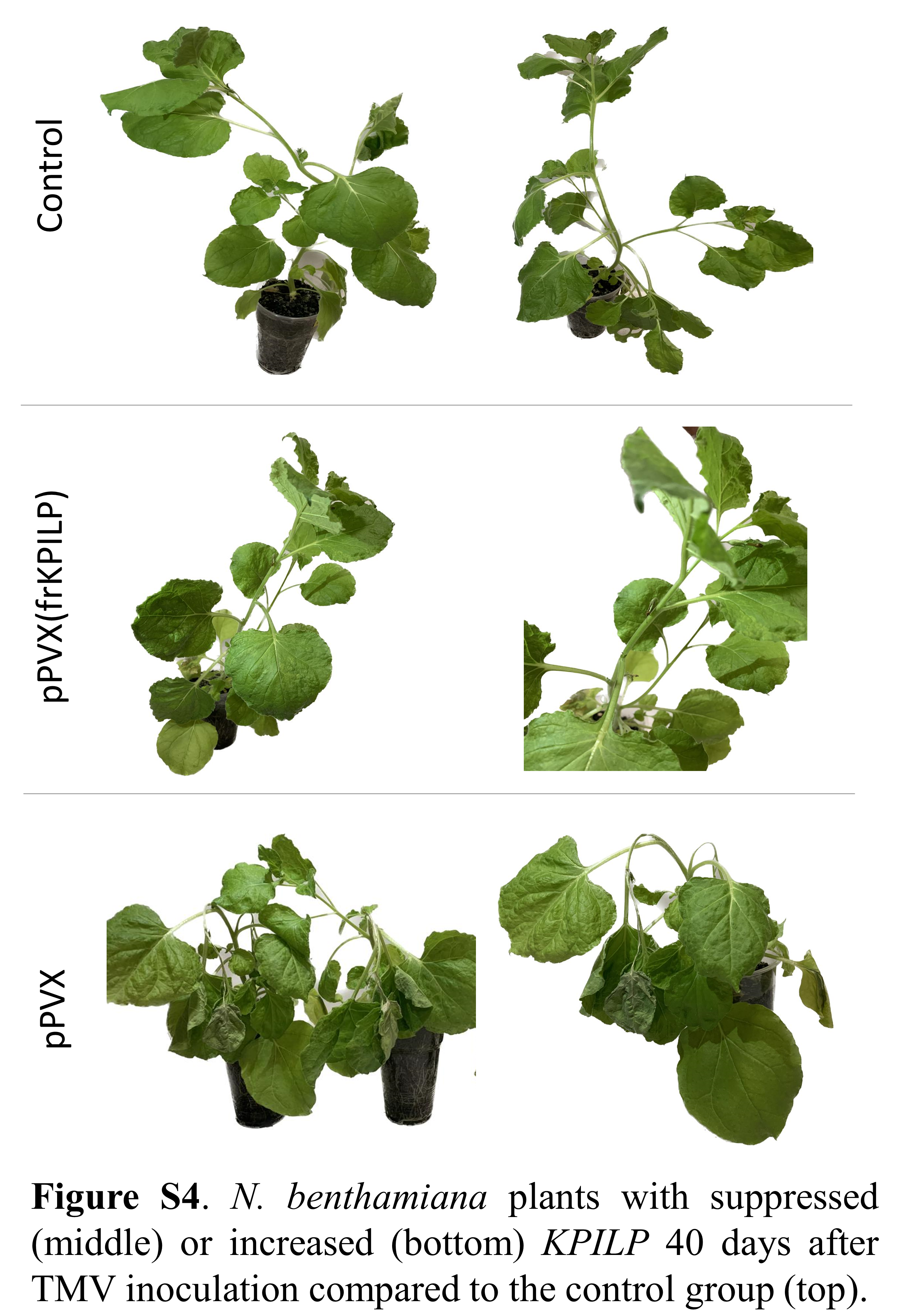

Supplement: Supplementary file 4 [file Image_4.tif]
