# Supplementary material for: A novel cellular factor of Nicotiana benthamiana susceptibility to tobamovirus infection
Source: Front Plant Sci. 2023 Jul 18;14:1224958. doi: 10.3389/fpls.2023.1224958 (PMC10390835; doi:10.3389/fpls.2023.1224958)
Supplement: Supplementary file 5 [file Table_1.docx]

**Table S1**. **Oligonucleotides used for qRT-PCR and cloning**

| Gene | Forward primer | Reverse primer |
| --- | --- | --- |
| *18S rRNA* | ACGGCTACCACATCCAAG | ACTCATTCCAATTACCAGACTC |
| *PP2A* | ATTGCTGCCTGTGGTTATTAC | ATAGACTGAAGTGCTTGATTGG |
| *KPILP* | TGAGCACTGGCGGAATTAAGG | ATACCAATATACCCACACAACAATCTG |
| **KPILP^[[1]](#footnote-1)^* | TCCGTCCAGGTTACACCTACT | CTTGAAAAACGTCGAGCGGG |
| *GLK* | GTCTCACTCGCCATAACATAGC | TTTCCGCCACCTCCAACC |
| *LHCB1* | TGTCGCCAAACCCGTCGCATCT | TTCACCGGTCAAGTAACTTGGG |
| *LHCB2* | TGCGACGTACGGTTAGAAGT | CTCGGAGAATGGTCCCAAGT |
| *RBCS1A* | GCTGCCTCATTCCCTGTTTC | CCTGCATGCATTGCACTCTT |
| *HEMA1* | ATGTGGGTGCTTGTGTGAAC | AGGCGGTCCTCCTTATTAGC |
| *RCA* | CATCTGTTGCCTCTTCATC | GCGTTCTCTTGCTTGTTG |
| *AtpC* | GCTCTTGGCATTGATTACAC | GTCAACCTCTTCACTCACG |
| *MP* TMV | GGTGTGAGCGTGTGTCTGG | GCGTCCTGGGTGGTTATAGC |
| *MP* crTMV | CGCAGCCAAGAGTAAGAG | GTGACCATAGCAACTGAAAC |
| *GFP* | GCAGAAGAACGGCATCAAG | GCTCAGGTAGTGGTTGTCG |
| siKPILP (sense) | CTCGAGTTCAAGTGCAGGGCTAGTAATTGACG | GAATTCAACCTTCTTGAACACAATCTTGAAAACTGG |
| siKPILP (antisense) | GGATCCAACCTTCTTGAACACAATCTTGAAAACTGG | TCTAGATTCAAGTGCAGGGCTAGTAATTGACG |

1. This pair of primers used only in experiments with 35S-siKPILP construct results of which are presented in Fig. 5. [↑](#footnote-ref-1)
